# Health- and social care in the last year of life among older adults in Sweden

**DOI:** 10.1186/s12904-020-00598-x

**Published:** 2020-06-23

**Authors:** Jenny Hallgren, Linda Johansson, Christina Lannering, Marie Ernsth Bravell, Catharina Gillsjö

**Affiliations:** 1 School of Health Sciences, University of Skövde, Skövde, Sweden; 2 Institute of Gerontology, School of Health and Welfare, Jönköping University, Jönköping, Sweden; 3 Futurum, Ryhov, Region Jönköping County, Jönköping, Sweden; 4 College of Nursing, University of Rhode Island, Kingston, USA

**Keywords:** Last year of life, Hospitalization, Home care services, National Quality Registries, Older adults

## Abstract

**Background:**

In the last years of life, burden of disease and disability and need of health- and social care often increase. Social, functional and psychological factors may be important in regard to social- and health care utilization. This study aims to describe use of health- and social care during the last year of life among persons living in ordinary housing or in assisted living facilities.

**Methods:**

A retrospective study examining health- and social care utilization during their last year of life, using a subsample from the Swedish twin registries individually linked to several Swedish national quality registries (NQR). Persons that died during 2008–2009 and 2011–2012 (*n* = 1518) were selected.

**Results:**

Mean age at death was 85.9 ± 7.3 (range 65.1–109.0). Among the 1518 participants (women *n* = 888, 58.5%), of which 741 (49%) were living in assisted living facilities and 1061 (69.9%) had at least one hospitalization during last year of life. The most common causes of death were cardiovascular disease (43.8%) and tumors (15.3%). A multivariable logistic regression revealed that living in ordinary housing, younger age and higher numbers of NQR’s increased the likelihood of hospitalization.

**Conclusions:**

Persons in their last year of life consumed high amount of health- and social care although 12% did not receive any home care. Married persons received less home care than never married. Persons living in ordinary housing had higher numbers of hospitalizations compared to participants in assisted living facilities. Older persons and persons registered in fewer NQR’s were less hospitalized.

## Background

In the last years of life, burden of disease and disability, and in turn, need of services and support from the society increase. For most persons the end of life is a period associated with care needs, hospitalizations, nursing home and hospice visits [1–3]. In Sweden, all citizens are entitled to health- and social care based on care needs and mainly financed by local taxes. During the last decades, the “stay in place” principle have influenced the home care policies in Sweden, meaning that older persons should live in their ordinary housing as long as possible, cared for by social services and home health care [4]. As a result, many persons with multiple illnesses remain in their ordinary housing at the same time as the years of life living in special accommodation i.e. assisted living facilities have decreased and persons living in these settings are older and frailer [5].

Several studies are indicating that the use of health care increases in the last year of life [6–8], although old age has been shown to be protective against hospitalization at the end of life [1]. Prior research has also shown that there is a divergent use of formal care in regard to gender and marital status. Women are more likely to live in assisted living facilities, and men are more likely to be hospitalized [9]. Since it is known that housing situation are important in regard to social- and health care utilization, comparisons of such may be helpful in developing supportive programs for persons living in assisted living facilities vs those having home care services at the end of life. Yet, care needs at the end of life may be measured in different aspects. In Sweden, the health care organizations providing primary health care, municipal care and care in hospitals are registering persons suffering from specific diseases and conditions in national quality registries. A national quality register in Sweden is defined as a registry that “contains individualized data concerning patient problems, medical, interventions and outcomes after treatment” and is often focused on a specific treatment of a disease [10]. The registries provide information to health care personnel, policy makers, patients and their relatives, of the incidence, treatments, symptoms, and different aspects of living with a specific disease and/or condition. Swedish national quality registries can also act as resources for comparative effectiveness research as they can be linked with nation-wide registries and health databases [11]. Quality registries aim to improve delivery of care but may also be a marker of burden of disease and subsequent health care needs [10, 12]. Since Sweden, and other western countries are struggling to allocate public resources as wisely as possible, it is of interest to know the amount of consumed health- and social care during the last year of life. This study aims to describe use of health- and social care during the last year of life among older persons living in ordinary housing or in assisted living facilities.

### Specific research questions

To what extent and for which conditions do older persons consume health- and social care during their last year of life?

Are there similarities in characteristics among persons in need of hospital care during their last year of life in regard to use of home care services, living conditions and marital status?

## Methods

### Study population and data collection

The present study is a part of the project; “Health Development in Late Life”. This is a project in which data from the Screening Across Lifespan Twin Study (SALT) [13], a subsample from the Swedish Twin Registries (STR), in 2014 were individually linked to several national quality registries (NQR) using the Swedish civic registration system. The registries included, comprise data of older persons and measures of their health and functional status collected through Senior Alert, Swedish Web-system for Enhancement and Development of Evidence-based care in Heart disease (Swedeheart), Swedish Dementia Registry (Swedem), Swedish Diabetes Registry (NDR), Swedish Heart Failure Registry (RiksSvikt), Swedish National Hip Fracture Registry, Swedish Stroke Registry (Riks-Stroke), Swedish Rheumatology Quality Registry (SRQ) [11] as well as the National Patient Registry (NPR), the Cause of Death Registry and the Care and Social services for older adults and for persons with impairments (SOL-registry). Twins in Sweden born before 1958, were in 1998 invited to participate in a telephone survey, which resulted in 44,816 participants in the SALT-study. Since SALT includes individual person data and is a representative sample of Swedish older persons, it was used as the basis for recruiting participants to this present study. After receiving ethical approval, applications where sent to the STR, National board of health and welfare and all national quality registries included to collect data. After receiving approvals, the National board of health and welfare conducted the matching process and sent anonymous data sets to the researchers. Each unique person had an identification number which made it possible to match different data sets on an individual basis. Information about the registries regarding coverage and content, are presented in Supplementary Table 1. In the present study, participants that had died during 2008–2009 and 2011–2012 and reached the age of 65 years (*n* = 1518) were selected. Participants that had died during 2010 was excluded since the SOL-registry did not provide sufficient data during 2009.

### Measurements

Data were collected in several patient and quality registers. The exact death date and cause of death according to the World Health Organization’s ICD-10 (International Classification of Diseases) were collected from the Cause of Death Registry. The diagnoses that were registered as causes of death were categorized into groups in accordance with the sections of the ICD [13]. Data regarding use of hospital care i.e. date of entry and discharge as well as the prevailing diagnoses during the last year of life, were obtained from the NPR. Hospitalization in this study meant at least one overnight stay. The SOL-registry provided information on use of social care services (help with personal care, “meals on wheels”, personal emergency response system (PERS), formal support to family and relatives, respite service to informal caregivers at home), care in institutional care facilities i.e. assisted living facilities, information on marital status (married/cohabiting, widowed/widower, divorced and never married) and whether a person lived in ordinary housing or in assisted living facilities.

### Statistical analyses

The χ2 test or t-test were used for comparing differences in participants´ characteristics and the use of social care (yes or no), living in ordinary housing or assisted living facilities, use of any social services i.e. help with personal care, “meals on wheels”, PERS, formal support to family and relatives, respite service to informal caregivers at home, and use of health care i.e. hospitalization (yes or no), numbers of hospitalizations, mean length of hospital stay, in terms of age, sex, marital status, number of quality registers and cause of death. The sample was further divided in age categories (65–75, 75–85, 85–90, > 90) and differences regarding use of home care services, hospitalizations and number of quality registers were compared in relation to age groups. Factors associated to hospital use by any cause in the last year, last 3 months and last month in life before death, were analyzed using a multivariable logistic regression model. Factors that included age, sex, marital status (married/cohabiting (reference category), widow/widower, never married or divorced), living conditions (ordinary home vs assisted living facilities), use of home care services (yes or no) and number of quality registers were entered simultaneously. A *p*-value < 0.05 was considered significant. All data was analyzed using SPSS Statistics 25.

### Ethics

This study was approved by Ethical Research Board in Linköping Sweden (Dnr 2014/2635–271).

## Results

Among the 1518 persons included (women *n* = 888, 58.5%), the mean age at death was 85.9 ± 7.3 (range 65.0–109.0) (women 86.7 ± 7.4, men 84.8 ± 7.0). Almost 50% of the sample 741 (48.8%), were living in assisted living facilities during their last year of life, and 706 (46.5%) were living alone. The number of persons living in ordinary housing were 291 (37.5%) and 415 (56.0%) lived in assisted living facilities. Among the married persons, 42 (5.9%) were living alone (7 in ordinary housing and 35 in assisted living facilities) and 113 (28.0%) of the married persons were cohabiting in assisted living facilities. Mean age at death was higher among those living in assisted living facilities than in ordinary homes, 87.3 years in comparison to 84.7 years of age. The majority were widow/widower in both settings. The mean age of the whole sample was higher for widow/widower (88.6), compared to being married (83.1), never married (83.7) or divorced (82.5) (Table 1). The most common causes of death were cardiovascular diseases (43.9%) and tumors (15.4%) (Table 2). It was more common for persons living in ordinary housing to have a death caused by tumors, compared to persons living in assisted living facilities in their last year of life. In contrast, persons dying of psychiatric conditions such as different types of dementia, were more likely to live in assisted living facilities in their last year of life.

**Table 1:** Descriptive characteristics

	Total sample *N* = 1518	Living in assisted living facilities (*n* = 741) % (n)	Living in ordinary housing (*n* = 777) % (n)	Comparisons between living conditions *p*	Home help services *n* = 649% (n)	No Home help *n* = 869% (n)	Hospitalized *n* = 1061% (n)	Not hospitalized *n* = 457% (n)	Age
Age mean (sd)	85.94 (7.3)	87.27 (6.89)	84.27 (7.47)	< 0.001	85.20 (7.24)	86.49 (7.32)	84.96 (7.24)	88.21 (6.96)	
Men % (n)	41.50 (630)	35.36 (262)	47.36 (368)	< 0.001	44.99 (292)	38.89 (338)	44.76 (475)	33.92 (155)	84.11 (6.96)
Women % (n)	58.50 (888)	64.64 (479)	52.64 (409)	< 0.001	55.01 (357)	61.11 (531)	55.23 (586)	66.08 (302)	86.74 (7.45)
*Marital status % (n)*									
Married	26.61 (404)	19.97 (148)	32.95 (256)		29.28 (190)	24.63 (214)	28.37 (301)	22.54 (103)	83.10 (6.69)
Widow/widower	50.92 (773)	57.76 (428)	44.40 (345)	< 0.001	47.15 (306)	53.74 (467)	48.07 (510)	57.55 (263)	88.69 (6.03)
Never married	10.61 (161)	10.66 (79)	10.55 (82)		10.63 (69)	10.59 (92)	11.22 (119)	9.19 (42)	83.68 (8.54)
Divorced	11.86 (180)	11.61 (86)	12.10 (94)		12.94 (84)	11.05 (96)	12.35 (131)	10.72 (49)	82.52 (7.92)
Hospitalization (Yes) % (n)	69.90 (1061)	49.93 (370)	88.93 (691)	< 0.001	68.52 (936)	81.52 (150)			84.96 (7.24)
Numbers of hospitalizations mean (sd)	3.04 (2.46)	2.42 (2.06)	3.36 (2.59)	< 0.000	3.02 (2.39)	3.17 (2.90)			
Mean length of stay mean (sd)	8.44 (9.57)	8.39 (8.14)	9.47 (11.65)	0.232	9.54 (12.08)	8.56 (8.33)			
No registration in quality register % (n)	32.67 (496)	30.23 (224)	35.01 (272)	0.047	34.82 (226)	31.10 (270)	29.50 (313)	40.04 (183)	84.96 (7.24)
Numbers of registers mean (sd)	1.12 (1.02)	1.14 (1.00)	1.11 (1.04)	0.361	1.09 (1.03)	1.14 (1.01)	1.22 (1.05)	0.89 (0.90)

**Table 2:** Causes of death

	All *N* = 1518 % (n)	Assisted living facilities % (n)	Ordinary housing % (n)	Not Hospitalized % (n)	Hospitalized % (n)
Certain infectious and parasitic diseases	2.04 (31)	2.29 (17)	1.80 (14)	0.88 (4)	2.54 (27)
Neoplasms	15.28 (232)	7.83 (58)	22.65 (176)	4.81 (22)	19.98 (212)
Blood and Endocrinological diseases^a^	3.75 (57)	3.38 (25)	4.12 (32)	3.28 (15)	3.96 (42)
Mental and behavioural disorders	10.01 (152)	16.87 (125)	3.47 (27)	20.57 (94)	5.47 (58)
Diseases of the nervous system	5.67 (86)	8.64 (64)	2.83 (22)	9.85 (45)	4.24 (41)
Diseases of the eyes, ears, nose and throat^b^	0.07 (1)	0	0.13 (1)	0	0.09 (1)
Diseases of the circulatory system	43.87 (666)	44.26 (328)	43.50 (338)	47.70 (218)	42.22 (448)
Diseases of the respiratory system	8.70 (132)	7.15 (53)	10.17 (79)	4.16 (19)	10.65 (113)
Diseases of the digestive system	2.24 (34)	2.16 (16)	2.32 (18)	0.66 (3)	2.92 (31)
Diseases of the skin and subcutaneous tissue	0.26 (4)	0.13 (1)	0.39 (3)	0	0.38 (4)
Diseases of the musculoskeletal system and connective tissue	0.46 (7)	0.54 (4)	0.39 (3)	0.66 (3)	0.38 (4)
Diseases of the genitourinary system	1.32 (20)	1.10 (8)	1.54 (12)	0.66 (3)	1.60 (17)
Symptoms, signs and abnormal clinical and laboratory findings, not elsewhere classified	3.75 (57)	4.18 (31)	3.35 (26)	5.91 (27)	2.83 (30)
Other disorders^c^	2.44 (37)	1.48 (11)	3.35 (26)	0.86 (4)	3.11 (33)
Total		741	777	457	1061

^a^Include Diseases of the blood and blood-forming organs and certain disorders involving the immune mechanism (*n* = 8) and Endocrine, nutritional and metabolic diseases (*n* = 49)

^b^Include Diseases of the eye and adnexa (*n* = 0) and Diseases of the ear and mastoid process (*n* = 1)

^c^Include Pregnancy, childbirth and the puerperium (*n* = 0), Certain conditions originating in the perinatal period (*n* = 0), Congenital malformations, deformations and chromosomal abnormalities (*n* = 0), Injury, poisoning and certain other consequences of external causes (*n* = 36), External causes of morbidity and mortality, Factors influencing health status and contact with health services (*n* = 1), Codes for special purposes

Among the 1518 persons included, 649 persons (42.8%) had care from the social services. Among those, 482 (74.3%) had help with services, 489 (75.3%) help with personal care, 79 (12.2%) had escort, 269 (41.5%) had help from “meals on wheels”, 484 (74.6%) had PERS, 5 (0.7%) support to family and relatives, and 38 (5.9%) had help with respite service to informal caregivers at home. Both men and women that received home care services were older than those not having home care. When dividing the sample in marital status, the married participants received less social care, compared to widow/widower, divorced and never married. Married women on the other hand, received more home care compared to men in their last year of life.

During the last year of life, 1061 (69.9%) participants had at least one hospitalization. Mean age of hospitalized participants was lower (84.9) than for those who had not been hospitalized (87.2). Some had up to 22 visits (mean 3.0, median 2.0) and mean length of stay in hospital was 8.4 days. Men had higher numbers of hospitalization days compared to women. Participants that did not receive home care were more frequently hospitalized compared to those having some kind of home care services. Being a widow/widower was associated with the least likelihood of being hospitalized compared to married, never married and divorced. Separating the sample based on housing revealed that participants living in ordinary housing were more likely to have experienced hospitalization, and they also had higher numbers of hospitalizations compared to participants in assisted living facilities (Table 3).

**Table 3:** Home help consumption and hospitalization divided in age groups

	65–75 (*n* = 119)	75–85 (*n* = 460)	85–90 (*n* = 430)	> 90 (*n* = 509)
Age, mean (sd)	70.20 (2.93)	80.62 (2.78)	87.18 (1.45)	93.37 (2.91)
No home help, % (n)	20.20 (24)	14.80 (68)	11.20 (48)	7.10 (36)
No hospitalizations, % (n)	15.10 (18)	23.00 (106)	28.40 (122)	41.50 (211)
Numbers of hospitalization all, mean (sd)	3.52 (3.08)	2.68 (2.84)	1.99 (2.21)	1.36 (1.79)
Numbers of hospitalization men, mean (sd)	3.89 (3.56)	2.73 (2.79)	2.13 (2.40)	1.76 (1.95)
Numbers of hospitalization women, mean (sd)	2.53 (0.32)	2.64 (2.88)	1.89 (2.05)	1.17 (1.68)
Numbers of hospitalization ALF, mean (sd)	2.46 (2.90)	1.55 (2.28)	1.24 (1.84)	0.82 (1.32)
Numbers of hospitalization OH, mean (sd)	4.00 (3.05)	3.50 (2.92)	2.72 (2.30)	2.13 (2.06)
Numbers of registers in quality registry, mean (sd)	1.11 (1.10)	1.15 (1.03)	1.20 (1.02)	1.04 (0.99)

*ALF* Assisted living facilities, *OH* Ordinary housing

**Fig. 1:**
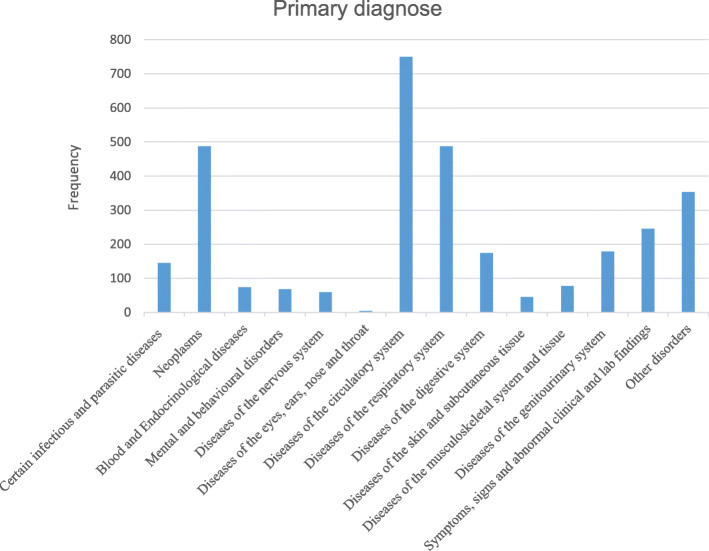
Primary diagnoses of all hospitalizations

**Fig. 2:**
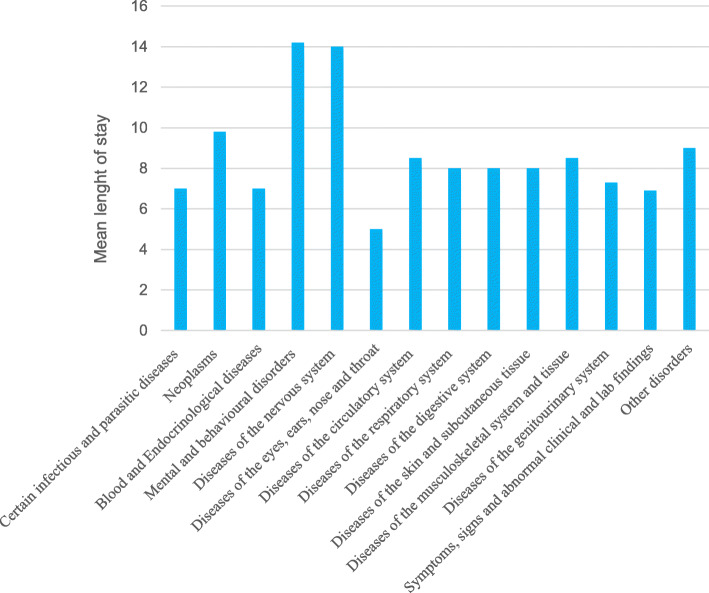
Mean length of stay in hospitals divided in primary diagnose during the last year of life

**Fig. 3:**
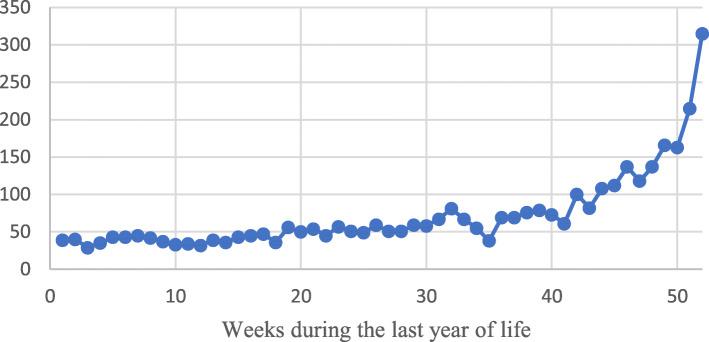
Numbers of participants in hospitals per week during their last year of life

The most common primary diagnoses among all participants that were hospitalized in the last year of life, were cardiovascular diseases, respiratory diseases (including pneumonia) and tumors (Fig. 1). The mean length of stay for all hospitalizations was 8 days, while the length of stay in hospital for psychiatric disorders and neurological disorders were about 15 days (Fig. 2). During the first 40 weeks in the last year of life, there were less than 80 persons hospitalized. However, during the last 10 weeks in life the numbers of persons being hospitalized increased to more than 150 (Fig. 3).

In the sample as a whole the mean number of registries in quality registers were 1.1. One third of the sample, 35% of the persons living in ordinary housing and 30% of those living in assisted living facilities, were not registered in any quality registries during their last year of life. Participants in assisted living facilities had higher mean numbers of registries compared to participants living in ordinary housing, but the difference was not statistically significant (Table 1).

When dividing the sample in age categories (65–75, 75–85, 85–90, > 90), persons between 65 and 75 years of age in both ordinary housing and assisted living facilities, were the most frequent consumers of hospitalization days in their last year of life. In contrast, the least days of hospitalization were among men and women in the oldest age group, 90 years and over. When stratifying age groups on gender, men were hospitalized to a greater extent in every age category, except in the youngest age group.

The multivariable logistic regression revealed that the likelihood of hospitalization increased with higher numbers of registries in NQR’s, in the last year of life, last 3 month as well as last month in life. Living in ordinary housing increased the likelihood of hospitalization in the last year and in the last 3 months in life. Younger age increased the likelihood of hospitalization for the last year of life, but not for the last 3 months or for the last month in life (Table 4).

**Table 4:** Multivariable Logistic regression of hospitalization risk, during last month and last 3 month of life

	Last year of life^a^			Last three months of life^b^			Last month of life^c^		
	B (SE)	*p*	Odds ratio	B (SE)	*p*	Odds ratio	B (SE)	*p*	Odds ratio
Age	−0.055 (0.010)	< 0.000	0.947	−0.011 (0.010)	0.268	0.989	−0.008 (0.013)	0.520	0.992
Sex (male)	0.263 (0.143)	0.066	1.301	−0.143 (0.151)	0.343	0.867	−0.101 (0.207)	0.290	0.862
Married/cohabiting	1	1	1	1	1	1	1	1	1
Widow/widower	−0.336 (0.242)	0.164	0.715	−0.003 (0.240)	0.990	0.997	0.422 (0.329)	0.199	1.524
Never married	0.034 (0.219)	0.878	1.034	−0.035 (0.229)	0.880	0.966	0.397 (0.318)	0.213	1.487
Divorced	0.064 (0.285)	0.821	1.066	0.098 (0.285)	0.754	1.093	0.756 (0.365)	0.038	2.130
Ordinary home	2.024 (0.304)	< 0.001	7.572	0.886 (0.256)	0.001	2.426	0.955 (0.319)	0.003	2.598
Home help	0.118 (0.317)	0.711	0.889	−0.249 (0.237)	0.294	0.780	−0.260 (0.288)	0.367	0.771
Numbers of registers	0.444 (0.068)	< 0.001	1.559	0.187 (0.065)	0.004	1.206	0.132 (0.080)	0.102	1.141

^a^*R*^2^ = 0.210 (Cox & Snell), 0.219 (Nagelkerke), Model x2 = 1481.742, *p* = <.001

^b^*R*^2^ = 0.047 (Cox & Snell), 0.077 (Nagelkerke), Model x^2^ = 1351.647, *p* = <.001

^c^*R*^2^ = 0.033 (Cox & Snell), 0.068 (Nagelkerke), Model x^2^ = 975.351, *p* = <.001

## Discussion

In this study social- and health care utilization in the last year of life among Swedish older adults were explored. Differences in care consumption were seen among participants living in assisted living facilities compared to ordinary housing. On average, persons that received care from social services or were living in assisted living facilities, were less hospitalized and had less numbers of hospitalizations, which support previous research [1, 14]. It is more likely that persons living in assisted living facilities receive professional health care and therefore avoid hospital admissions [14]. In this study 70% were hospitalized in their last year of life and had higher numbers of hospitalizations when approaching death. Pivodic et al. [6] found the same pattern of an increased likelihood of being hospitalized in the last month of life in four different European countries that were included in their study. Many countries aim to reduce end of life hospitalizations but the policies for end of life care differ substantially [15]. According to a Swedish report comparing health care in ten different countries, Sweden has low ranking in planning of care for end of life [16]. Lack of discussions and/or agreements between patients and health care personnel regarding treatments, may be one reason for the higher degree of hospitalization for persons living in ordinary housing, as seen in this study.

Additionally, the study showed that consumption of home care where related to age. Participants, both men and women, receiving home care were older than those not having services from home care, which is similar to findings in earlier Swedish research showing that the majority of the oldest old use formal care service, home care or institutional care, at the end of their lives [3, 7, 17]. Older age has been shown to be a risk factor of hospitalization [18] and readmission [19]. However, during the last year of life, older age was not associated with hospitalization in this study, which is in line with prior research [1, 20]. It is possible that staff in assisted living facilities, home health care and primary health care are gatekeepers and prevent that older persons are hospitalized in their last days in life, and care for them in their ordinary housing which is congruent with health – and social care policies. As the aging population increases, changes in health policies that promote the transfer of health care from formal places such as hospitals and institutions to the more informal setting of one’s home is needed [21]. However, research is inconsistent in regard to preferences about the desire to die at home or in institution [22, 23], which addresses the importance of a person centered care in which these matters are prioritized.

When stratifying on marital status, the married participants received less home care, compared to persons being widow/widower, never married and divorced. However, among the married persons, women received more home care than men in their last year of life. Men on the other hand, were consuming a higher number of hospitalization days in every age group, which support previous research [1, 6]. It has been found that older, single living men in particular are at risk of being hospitalized to a greater extent than women [24]. Earlier research has shown that women in late middle and older age have worse functioning and disability problems than men, but these problems are rarely major causes of mortality or hospitalization [25]. Further research with in-depth analyses of health care consumption in regard to gender and marital status differences are warranted.

Interestingly, there were different utilization of health- and/or social services in the last year of life depending on the cause of death. Persons that died of tumors were more likely to live in ordinary housing in their last year of life, while persons dying of psychiatric disorders were more likely to live in assisted living facilities. It can to some extent be assumed that persons suffering from tumors are cared for by their relatives. Yet, in this study very few received formal support to family and relatives or respite service for informal care givers at home. This can be viewed in light of the commonness of unfinanced informal care givers, not seldom a preference and strive to provide palliative care at home in a private, comfortable and safe environment, instead of in an institution [26, 27]. However, in this study hospitalizations due to psychiatric and neurological disorders, demanded a higher number of hospital days compared to other reasons for hospitalization. It was in average less days spent in hospitals in this current study compared to other countries in Europe where it has been reported hospitalization 1 month or more in inpatient facilities at hospitals during the last year of life. Persons from the east and south of Europe were more likely to be hospitalized compared to northern and eastern Europe, including Sweden [1].

No difference was found in living conditions or use of services from home care, and numbers of quality registries in the descriptive analyzes. However, when entering numbers of registries in a multivariable logistic regression, a higher number of quality registries were associated with the number of hospitalization in the last year, the last 3 months as well as the last month in life. These results may imply that higher numbers of registries in quality registers indicate the severity of illness, and predict need of health care during end of life. However, most registries in NQR’s occur in the hospital setting [12], and it can be expected that the registries in NQR’s may increase along with higher numbers of hospitalizations. Yet, the result of this study contribute to knowledge and inspiration on how research using NCR’s individually merged to nation-wide patient data, can improve end of life health care planning.

The strength in this study is the unique clinical data were several patient registers and quality registers were individually merged together, allowing unique potentials in analyzing patient characteristic and health- and social care utilization. Furthermore, the sample drawn from the STR, has in previous studies shown to be representative for the Swedish population. However, as for all research projects, this study has limitations. For instance, there was no information of place of death, and only data on health- and social services based on the last six-months. It would have been interesting to follow this type of information on a week to week basis, allowing deeper analyzes of changes related to level of services from home care and care in the context of assisted living facilities during the last year of life. Furthermore, information on numbers of NQR’s should be carefully interpreted since we included eight of several eligible NQR’s in Sweden, and the coverage rate is low in some of the registries.

## Conclusion

To conclude, persons in their last year of life consume a high amount of health- and social care although as many as 12% did not receive any home care services. Married persons received less home help than widow/widower, never married and divorced. Persons living in ordinary housing were more likely to have experienced hospitalization and they also had higher numbers of hospitalizations compared to participants in assisted living facilities. Men were more frequently hospitalized compared to women in all age groups over 65 years of age. Older persons and persons registered in fewer NQR’s were less likely hospitalized.

## Supplementary information

**Additional file 1: Table S1.** Description of the national health registers and NQRs included.

## Data Availability

Datasets from this study are not available since we do not have the consent to share the data neither from the Ethical Research Board nor from the participants.

## Abbreviations

SALT: Screening Across Lifespan Twin Study
STR: Swedish Twin Registries
NQR: National quality registries
Swedeheart: Swedish Web-system for Enhancement and Development of Evidence-based care in Heart disease
Swedem: Swedish Dementia Registry
NDR: Swedish Diabetes Registry
RiksSvikt: Swedish Heart Failure Registry
Riks-Stroke: Swedish Stroke Registry
SRQ: Swedish Rheumatology Quality Registry
NPR: National Patient Registry
SOL-registry: Care and Social services for older adults and for persons with impairments
ICD: International Classification of Diseases
PERS: Personal emergency response system
