# Supplementary material for: Health- and social care in the last year of life among older adults in Sweden
Source: BMC Palliat Care. 2020 Jun 23;19:90. doi: 10.1186/s12904-020-00598-x (PMC7313197; doi:10.1186/s12904-020-00598-x)
Supplement: Supplementary file 1 — Additional file 1: Table S1. Description of the national health registers and NQRs included. [file 12904_2020_598_MOESM1_ESM.docx]

| **SupplementaryTable 1. Description of the National health registers and NQRs included** | | | | | |
| --- | --- | --- | --- | --- | --- |
| **Register**  **(Short name)** | **Purpose** | **Launched** | **Completeness (for data 2014)** | **Content** | **Users** |
| **Health and care registers** | | | | | |
| Cause of Death registry | To be able to describe the causes of death and to follow the development of mortality in Sweden. | 1961 | 100% | Variables about death date, place, causes of death including accidents and diseases | Physicians |
| National patient registry (NPR) | To monitor the health development of the Swedish population over time, improve the possibilities of preventing and treating diseases, contribute to the development of health care and, monitor the quality of health care. | 1964 | 100% | Variables covering care contacts, diagnosis, external cause for disease and death and interventions. | Physicians |
| Care and social services for the elderly and for the persons with impairments (SOL-registry) | Provides information for official statistics on social care. | 2007 | Not fully known but estimated as high for all years except for the years 2009 and 2013. | Number of hours and type of care including home-help, short-term stays, day care, and nursing home placement. Specific type of home-help including personal care, escort, meals-on-wheels, support to informal caregivers and personal emergency response system. | Data received from records at each municipality |
| **National quality registers** | | | | | |
| Swedish Heart Failure Registry  (RiksSvikt) | To improve treatment of patients with heart failure and, identify changes in the quality of care and content over time in county councils and health care units. | 2003 | 48,9%^1^. | Variables covering information about type of care, diagnosis, symptoms, treatment, lab tests and exams, quality of life, depression, alcohol, smoking and living situation. | Patients, professionals |
| Swedish Web-system for Enhancement and Development of Evidence-based care in Heart disease  (Swedeheart)  Includes four registers:  RIKS-HIA (Swedish Registry of Information and Knowledge about Swedish Heart Intensive Care Admissions), SCAAR (Swedish Coronary Angiography and Angioplasty Registry), heart surgery registry and SEPHIA (Secondary Prevention after Heart Intensive Care Admission) which is part of RIKS-HIA. | To support the development of evidence-based therapy in acute and chronic coronary artery disease and in catheter-based or surgical valve intervention. To detect changes in care content and quality to contribute to the advancement of risk prediction tools and decision support, as well as improvement efforts. | 2009 | RIKS-HIA^2^ 83,5%  SCAAR^2^ 98,9%.  Heart surgery registry^2^ 96,8%. | Variables covering information about type of care, diagnosis, symptoms, treatment, lab tests and exams, physical activity, smoking, quality of life, life style and occupation. | Patients, professionals |
| Swedish Stroke registry (Riks-Stroke) | To support high and consistent quality of care for stroke patients throughout Sweden, ultimately to ensure patient benefit in the form of the best possible care. | 1994 | 87,6%^2.^ | Variables covering information about type of care, diagnosis, treatment, symptoms, depression, cognition, nursing aspects, ADL, smoking, living situation and home-help.^.^ | Patients, professionals |
| Swedish diabetes registry (NDR) | To reduce morbidity and mortality, as well as to maximize the cost-effectiveness of diabetes care by promote evidence-based development and improve the diabetes care. | 1996 | 75,6%^2^. | Variables covering information about type of care, diagnosis, lab tests and exams, physical activity, smoking and treatment. | Patients, professionals |
| The Swedish Rheumatology Quality Registry (SRQ) | To continually improve treatment and follow-up of patients with rheumatic diseases. | 1996 | 74,8%^2^. | Variables covering information about type of care, diagnosis, treatment, symptoms, ADL, quality of life, lab tests and occupation. | Patients, professionals |
| Swedish National Hip Fracture Registry (Rikshöft) | To report performance measures to compare, improve and create consistent and high-quality care. | 1988 | 82,0%^2^. | Variables covering information about type of care, diagnosis, treatment, symptoms, ADL, , quality of life, smoking, alcohol, exams, cognition, occupation and living situation. | Patients, professionals |
| Swedish Dementia Registry (Svedem) | To improve quality of diagnostics, treatment and care of patients with dementia disorders. | 2007 | 43,4%^2^¤ | Variables covering information about type of care, diagnosis, treatment, ADL, quality of life, exams, cognition, occupation, living situation and home-help. | Professionals |
| Senior Alert | To ensure a preventative approach for persons over 65 years of age with care needs within the areas of   - falls - ulcers/pressure sores - malnutrition - oral health | 2010 | 27%^4^ | Variables covering information about type of care, exams and nursing care. | Professionals |

^1^Based on data from 2013 according to the reference <https://www.socialstyrelsen.se/globalassets/sharepoint-dokument/artikelkatalog/statistik/2014-12-7.pdf>

^2^Based on data from 2012 according to reference <https://www.socialstyrelsen.se/globalassets/sharepoint-dokument/artikelkatalog/ovrigt/2013-12-12.pdf>

^3^ Based n data from 2012 according to the reference <https://registercentrum.blob.core.windows.net/boa/r/BOA-rsrapport-2012-ByS8BlwWl.pdf>

^4^ Based on data from 2013 According to reference <https://www.socialstyrelsen.se/globalassets/sharepoint-dokument/artikelkatalog/ovrigt/2018-2-17.pdf>
